# Guest Editorial

**Published:** 2008-10

**Authors:** K. Ramachandran

**Affiliations:** Guest Editor, Consultant Cosmetic Surgeon, Apollo Hospital, Chennai, India

During the past decade there has been a significant increase in the aesthetic surgical procedures being performed in our country. This has been due to various factors. The concern for self image, the improving economy and also the availability of skilled professionals are some of them, to name a few. Though a variety of conditions with different surgical options are available, this issue aims at looking only at some of the common aesthetic procedures. The trend towards non-surgical or minimally invasive surgery in the management of early aging has been addressed along with reference to anti aging medicine. One need not be surprised that the land where plastic surgery originated has also been the home to many non-surgical options for beautification as has been reflected in the historical article on herbal cosmetics. Through this one can realize that even in ancient India recipes to tackle conditions like facial hair and anti aging creams were available. The discussion on Jacques Joseph is included as a backdrop to this issue and his contribution to modern aesthetic surgery truly will give an insight about this genius. The various authors who have contributed to this special issue have spared their valuable time in putting their thoughts together. I wish to thank each and every one of them for spontaneously agreeing to my request. I also wish to acknowledge the efforts put in by the editor Dr. Mukund Thatte and his team, in helping me bring out this special issue. This compilation aims at providing insight into some of the common aesthetic surgical problems and I sincerely hope that it fulfils this end.

